# Estrogen Action in the Epithelial Cells of the Mouse Vagina Regulates Neutrophil Infiltration and Vaginal Tissue Integrity

**DOI:** 10.1038/s41598-018-29423-5

**Published:** 2018-07-26

**Authors:** Shuai Li, Gerardo G. Herrera, Keila K. Tam, Jacob S. Lizarraga, My-Thanh Beedle, Wipawee Winuthayanon

**Affiliations:** 1 Department of Biochemistry and Molecular Biology, Mayo Clinic, Rochester, Minnesota 55902 USA; 2 School of Molecular Biosciences, Center for Reproductive Biology, College of Veterinary Medicine, Washington State University, Pullman, Washington 99164 USA

## Abstract

In the female reproductive tract, the innate immune system is modulated by two sex steroid hormones, estrogen and progesterone. A cyclical wave of neutrophils in the vaginal lumen is triggered by chemokines and correlates with circulating estrogen levels. Classical estrogen signaling in the female reproductive tract is activated through estrogen receptor α (encoded by the *Esr1* gene). To study the role of estrogen action in the vagina, we used a mouse model in which *Esr1* was conditionally ablated from the epithelial cells (*Wnt7a*^cre/+^; *Esr1*^f/f^). Histological evidence showed that in response to a physical stress, the lack of ESR1 caused the vaginal epithelium to deteriorate due to the absence of a protective cornified layer and a reduction in keratin production. In the absence of ESR1 in the vaginal epithelial tissue, we also observed an excess of neutrophil infiltration, regardless of the estrous cycle stage. The histological presence of neutrophils was found to correlate with persistent enzymatic activity in the cervical-vaginal fluid. Together, these findings suggest that ESR1 activity in the vaginal epithelial cells is required to maintain proper structural integrity of the vagina and immune response, both of which are necessary for protecting the vagina against physical damage and resetting the vaginal environment.

## Introduction

The female reproductive tract is composed of upper (oviduct, uterus, and endocervix) and lower (ectocervix and vagina) sections. Recent studies of the upper female reproductive tract (UFRT) suggest that estrogen receptor α (ESR1) in the epithelial cells is crucial for embryo development and transport in the oviduct^1,2^, as well as for semen liquefaction in the uterus^3^. ESR1 is required in epithelial cells of the lower female reproductive tract (LFRT) to regulate the differentiation of vaginal epithelial cells^4^. Several lines of evidence suggest that the vaginal epithelial cells may also act as a physical barrier to prevent both external damage and pathogen infiltration^5–7^. These activities can be mediated through ESR1, demonstrated by a study in which the conditional ablation of ESR1 from vaginal epithelial cells resulted in reduced epithelial thickness^4^. Additionally, altered levels of its endogenous ligand estrogen (E_2_) have been shown to differentially modulate the number of antimicrobial proteins and inflammatory genes that are expressed^7–9^. Despite this, the role of vaginal epithelial ESR1 in maintaining vaginal tissue integrity and immune response is currently a research question that has not been fully investigated.

The growth and differentiation of vaginal epithelial cells are regulated by the sex steroid hormones estrogen (E_2_) and progesterone (P_4_). In mice, the pattern of leukocyte infiltration corresponds with circulating E_2_ and P_4_ levels and is used to determine the stage of the estrus cycle^10^. The estrous cycle is divided into four distinct stages: estrus, metestrus, diestrus, and proestrus. Vaginal smears from mice in estrus stage contain mostly cornified cells, and mice at this stage are receptive to copulation^10^. At estrus, the P_4_ level is elevated but will gradually decline towards the end of the cycle. If mating does not take place, the cycle will continue towards the metestrus stage. At metestrus, a large number of leukocytes will infiltrate the vaginal lumen and clear out the cornified cell debris^11^. At this stage, circulating E_2_ returns to basal levels. Metestrus is followed by diestrus, a resting stage of the reproductive cycle, and vaginal smears from animals in diestrus show a reduced number of cell types. Towards the end of diestrus, E_2_ levels begin to rise, and the cycle progresses to proestrus. It is during proestrus that E_2_ and luteinizing hormone levels peak, restarting the cycle. Nucleated epithelial cells are the dominant cell populations observed in vaginal smears obtained from animals in proestrus^10^. Importantly, these different stages are accompanied by changes in vaginal secretory proteins such as cytokines, chemoattractants, and antimicrobial molecules^12^.

E_2_ signaling in the vagina induces the formation of a physical barrier by maintaining the thickness of the vaginal epithelium and increasing the secretion of antimicrobial peptides, cytokines and chemokines (which recruit and activate immune cells)^13–15^. The epithelial lining of the vagina is also covered by a layer of glycoprotein-containing mucus, namely mucins, that protects the vagina from infectious agents^16,17^. Vaginal epithelial cells are stimulated by E_2_ signaling to produce glycogen, a substrate that is metabolized by the native microflora^18^. In postmenopausal women, a reduction in E_2_ levels results in the production of fewer epithelial cells and a reduced glycogen content. With vaginal epithelial cell atrophy and a lack of lubrication, the vaginal tissue becomes vulnerable to physical damage, e.g. intercourse, and is subsequently susceptible to infection. Additionally, the composition of the vaginal microbiota often changes in postmenopausal women due to the reduction in the nutritional substrate for the microbiota^19,20^. These changes in the vaginal environment are often alleviated by treatments with a topical synthetic E_2_^21^, suggesting that E_2_ signaling within the vagina plays a role in the maintenance of the immune response and vaginal homeostasis. Although E_2_ supplementation is the current course of treatment for most postmenopausal symptoms in women, alternative non-hormonal treatment options are often desired. A better understanding of the E_2_-regulated actions in the vaginal epithelium is critical to the development of alternative therapeutic options for postmenopausal women.

The effects of E_2_ in the female reproductive tract are primarily mediated through its receptor ESR1. To assess the effects of ESR1 activity on the vaginal epithelium, Miyagawa and Iguchi utilized a keratin 5-driven Cre mouse model to genetically ablate *Esr1* from the vaginal epithelial cells (*Krt5*^cre/+^; *Esr1*^f/f^)^4^. Using this model, the authors showed a reduced thickness in the vaginal epithelium, thereby suggesting a role for ESR1 in controlling vaginal epithelial cell differentiation^4^. Although this data is informative, the changes imparted on the vaginal epithelium in the absence of ESR1 following physical stresses have never been investigated. In the absence of ESR1, physical stresses, especially those induced by mating, could exacerbate the observed effects on the vaginal epithelium. A compromised epithelial layer is likely to alter vaginal homeostasis, eliciting an observable immune response that differs from an individual with normal ESR1 function. Therefore, we hypothesized that ESR1 activity within the vaginal epithelial cells is not only required for the maintenance of vaginal tissue integrity, but is also involved in the regulation of the immune response following a physical stress.

To assess this, we generated a similar model in which ESR1 was deleted in the vaginal epithelium using the *Wnt7a*^cre/+^ (*Wnt7a*^cre/+^; *Esr1*^f/f^)^22^. Our *Wnt7a*^cre/+^; *Esr1*^f/f^ females ovulate normally without the need for an exogenous gonadotropins^3^. These *Wnt7a*^cre/+^; *Esr1*^f/f^ females are also receptive to copulation^3^, allowing us to validate the role of ESR1 in maintaining vaginal tissue integrity after physical stress *in vivo*. Additionally, we used this model to investigate the role of vaginal epithelial ESR1 for both neutrophil recruitment/infiltration and maintenance of the vaginal microbiota.

## Results

### Loss of ESR1 function in the epithelium induces vaginal tissue laceration after mating

**Figure 1:**
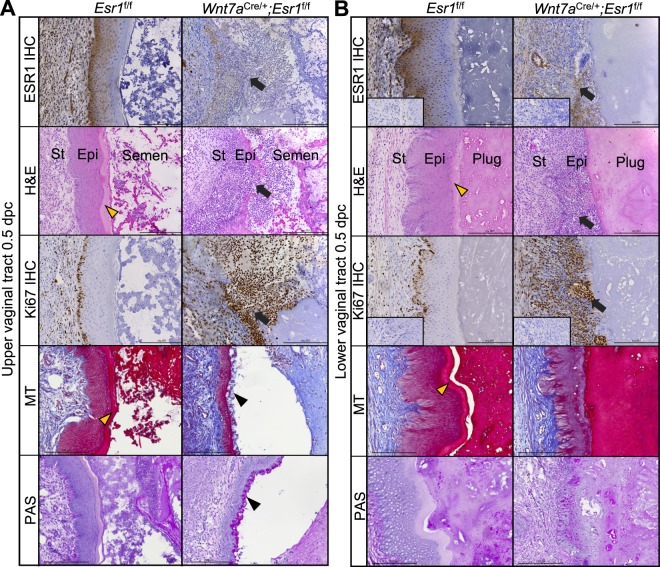
Loss of tissue integrity and leukocyte infiltration after mating in the absence of vaginal epithelial ESR1. Immunohistochemical (IHC) staining of ESR1, H&E, Ki67 IHC, Masson’s Trichrome (MT), and Periodic Acid-Schiff (PAS) staining in (**A**) the upper vaginal tract and (**B**) the lower vaginal tract, respectively, at 0.5 dpc in *Esr1*^f/f^ and *Wnt7a*^cre/+^; *Esr1*^f/f^ animals. Black arrows indicate a site of cell infiltration from stromal and epithelial layers into the vaginal lumen. Yellow arrowheads indicate the distinct cornified layer in *Esr1*^f/f^ tissues. Black arrowheads denote the PAS-positive cell on the apical side of *Wnt7a*^cre/+^; *Esr1*^f/f^ vaginal lumen. Insets are negative control without primary antibodies. Scale bars = 100 µm. *n* = 4–6 mice/genotype.

The physiological impact of mating in animals lacking ESR1 in vaginal epithelial cells has yet to be explored. Therefore, histological evaluation was performed on vaginal tissues at 0.5 days post coitus (0.5 dpc) in *Esr1*^f/f^ and *Wnt7a*^cre/+^; *Esr1*^f/f^ adult (8- to 12-week-old) female mice. First, ESR1 immunohistochemical (IHC) analysis was performed to ensure ablation of ESR1 in the vaginal epithelium. ESR1 protein was expressed in all cell layers of the *Esr1*^f/f^ vaginal tract (Fig. 1). In the vaginal tract of *Wnt7a*^cre/+^; *Esr1*^f/f^ animals, ESR1 protein was absent from the entire epithelial cell layer, whereas expression of ESR1 in the stromal layer remained intact (Fig. 1). Next, the vaginal histoarchitecture was evaluated using hematoxylin & eosin (H&E) staining. *Esr1*^f/f^ vaginal tracts showed well-defined and distinguished epithelial layers (Fig. 1). These included a cornified outer layer (containing enucleated cells, Fig. 1, yellow arrowheads), stratified epithelial cell layers, and stromal cell layers in both the upper and lower vaginal tract. In contrast, vaginal epithelial layers within the *Wnt7a*^cre/+^; *Esr1*^f/f^ animals were severely disorganized (Fig. 1). Representative images showed a disrupted epithelial layer with cells infiltrating from the stroma into the vaginal lumen (Fig. 1, black arrows).

**Figure 2:**
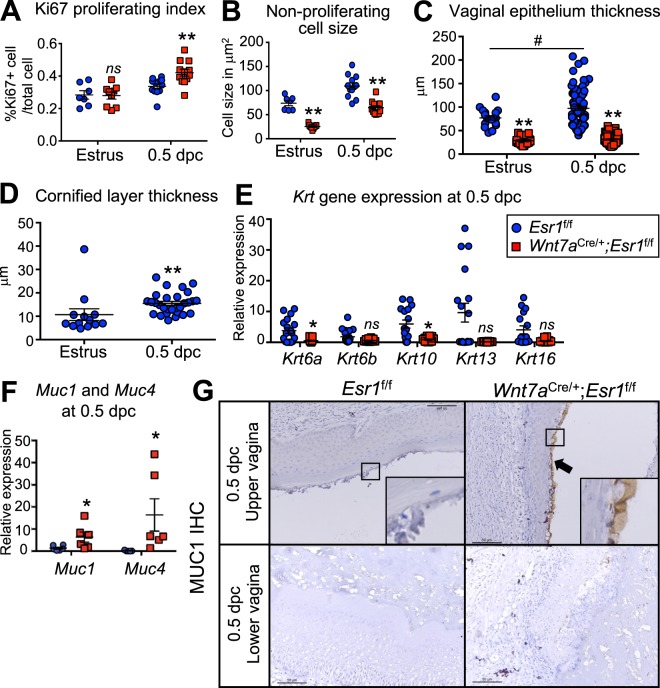
Vaginal proliferation index, epithelial thickness, keratinization, and mucification in *Esr1*^f/f^ compared to *Wnt7a*^cre/+^; *Esr1*^f/f^ animals. (**A**) Proliferating index of vaginal epithelial cells of *Esr1*^f/f^ and *Wnt7a*^cre/+^; *Esr1*^f/f^ animals at estrus and 0.5 dpc as indicated by the percentage of Ki67-positive cells relative to the total cell number. (**B**) Non-proliferating epithelial cell size in *Esr1*^f/f^ and *Wnt7a*^cre/+^; *Esr1*^f/f^ vaginal tissues indicated by Ki67-negative cells. Data presented as mean ± SEM. **p < 0.01 compared to *Esr1*^f/f^ at the similar time-point, two-way ANOVA. *ns* = not statistically significant. *n* = 6 mice/genotype. (**C**) The total thickness of the vaginal epithelium at estrus and 0.5 dpc stages. **p < 0.01 compared to *Esr1*^f/f^ at the similar time-point, two-way ANOVA. ^#^*p* < 0.05, compared between *Esr1*^f/f^ at estrus and 0.5 dpc, two-way ANOVA. *n* = 4–6 mice/genotype. (**D**) Thickness of the cornified layer in *Esr1*^f/f^ animals at estrus and 0.5 dpc (*p < 0.05, Mann-Whitney test, *n* = 4–6 mice/genotype). (**E**) Transcription levels of keratin gene family in the whole vaginal tissue at 0.5 dpc, indicated by RT-qPCR analysis (*p < 0.05, Mann-Whitney test, *n* = 5–6 mice/genotype). (**F**) *Muc1* and *Muc4* mRNA levels in the whole vaginal tissue at 0.5 dpc (*p < 0.05, Mann-Whitney test, *n* = 3 mice/genotype). (**G**) MUC1 IHC staining in the upper and lower vaginal tract of *Esr1*^f/f^ and *Wnt7a*^cre/+^; *Esr1*^f/f^ animals. Insets are the higher magnification of apical layer. Arrows denotes sat cells with MUC1-positive staining. *n* = 4–6 mice/genotype. Data presented as mean from each technical replicate ± SEM. *ns* = not statistically significant. Scale bars = 50 µm.

To determine whether proliferation was disrupted in the *Wnt7a*^cre/+^; *Esr1*^f/f^ vaginal epithelium after mating, we performed IHC analysis with Ki67 (a proliferative marker) at 0.5 dpc. Ki67-positive cells were detected at the basal layer of the epithelium and within the lumen of the *Esr1*^f/f^ vaginal tract (Fig. 1), whereas at sites with a disrupted epithelial cell layer in the *Wnt7a*^cre/+^; *Esr1*^f/f^ animals, all infiltrating cells were Ki67-positive. To determine the proliferative index, we evaluated the number of Ki67-positive cells present in vaginal tissues during estrus as well as at 0.5 dpc in areas without disruption sites. The proliferative index in the epithelial layer was not different at estrus, but was significantly higher at 0.5 dpc in *Wnt7a*^cre/+^; *Esr1*^f/f^ compared to *Esr1*^f/f^ controls (Fig. 2A). Because the epithelial layer contains both Ki67-positive and -negative cells in *Esr1*^f/f^ tissues (Fig. 1), the cell size of Ki67-negative cells was measured. We found that Ki67-negative cells in the epithelial cell layer were significantly smaller in *Wnt7a*^cre/+^; *Esr1*^f/f^ compared to *Esr1*^f/f^ controls at both estrus and 0.5 dpc (Fig. 2B).

To determine whether the keratin-rich cornified layer was also absent in *Wnt7a*^cre/+^; *Esr1*^f/f^ vaginal epithelium after mating, Masson’s Trichome (MT) staining was performed. A clear cornified layer was observed in the *Esr1*^f/f^ mice but no cornified layer was detected in the *Wnt7a*^cre/+^; *Esr1*^f/f^ tissue (Fig. 1, yellow arrowheads, MT staining). Consequently, the vaginal plug was observed to be directly adhered to the basal epithelium in the *Wnt7a*^cre/+^; *Esr1*^f/f^ lower vaginal tract (Fig. 1B, MT staining). In some areas, the semen/copulatory plug was also observed to be lodged between the detached epithelial surface layer and the basal layer (Supplementary Fig. S1), while in other areas, the epithelial cell layer was completely absent and the vaginal plug was adhered directly to the stromal cell layer (Supplementary Fig. S1). Interestingly, a blue staining layer indicative of glycogen content was observed at the apical surface in only the *Wnt7a*^cre/+^; *Esr1*^f/f^ vaginal tissues (Fig. 1A, black arrowhead). Periodic acid–Schiff (PAS) staining confirmed that a globular-shape glycoprotein (possibly mucin) was present at the apical surface of the *Wnt7a*^cre/+^; *Esr1*^f/f^ upper vaginal tract (Fig. 1A, black arrowhead). At estrus, intense PAS staining was also observed at the apical surface of the *Wnt7a*^cre/+^; *Esr1*^f/f^ vaginal tract (Supplementary Fig. S1).

### Epithelial ESR1 signaling is involved in *Krt* and *Muc* gene expression

In *Esr1*^f/f^ control mice, the whole vaginal epithelium thickness (is composed of both epithelial cells and a cornified layer) at estrus was 77.03 ± 18.82 μm (Fig. 2C). A 10.69 μm cornified layer contributed to this vaginal epithelium thickness (Fig. 2D). After mating, the whole vaginal epithelium thickness in *Esr1*^f/f^ controls was further increased to 97.41 ± 29.40 μm with an average 15.51 μm cornified layer (Fig. 2C,D). In contrast, *Wnt7a*^cre/+^; *Esr1*^f/f^ animals had a significantly thinner epithelial layer at estrus (28.55 ± 8.87 μm) and at 0.5 dpc (31.34 ± 7.52 μm, Fig. 2C). Due to a lack of a cornified layer in *Wnt7a*^cre/+^; *Esr1*^f/f^ animals, no measurements of the cornified layer in *Wnt7a*^cre/+^; *Esr1*^f/f^ animals could be made. As keratinization strengthens the epithelium we next determined the expression of the keratin family of genes, specifically *Krt6a*, *Krt6b*, *Krt10*, *Krt1*3, and *Krt16*. Only two of the tested keratin genes, *Krt6a* (a type 2 keratin) and *Krt10* (a type 1 keratin), were expressed at significantly lower levels in the *Wnt7a*^cre/+^; *Esr1*^f/f^ compared to *Esr1*^f/f^ vaginal tissues (Fig. 2E, *p* = 0.0426 and 0.0111, respectively).

To determine whether the glycoproteins indicated by the PAS staining in *Wnt7a*^cre/+^; *Esr1*^f/f^ vaginal tissues were mucins, the expression of mucin genes and presence of mucin proteins were also analyzed. *Muc1* and *Muc*4 genes were both expressed at significantly higher levels in *Wnt7a*^cre/+^; *Esr1*^f/f^ animals compared to *Esr1*^f/f^ controls (Fig. 2F, *p* = 0.026 and 0.0043 respectively). MUC1 protein was detected at the surface of the upper vaginal epithelium in *Wnt7a*^cre/+^; *Esr1*^f/f^ animals and was not detectable in *Esr1*^f/f^ controls (Fig. 2G). MUC1 protein was also detected at lower levels in some areas of the lower vaginal tract of *Wnt7a*^cre/+^; *Esr1*^f/f^ animals (Fig. 2G) compared to a non-detectable level in the controls. Mucosal MUC1 protein was present in both the uterus and endocervix of *Esr1*^f/f^ and *Wnt7a*^cre/+^; *Esr1*^f/f^ animals. However, the detection of MUC1 protein in *Esr1*^f/f^ animals was gradually decreased between the endocervix and ectocervix (Supplementary Fig. S2).

### Increased leukocyte infiltration in the absence of ESR1 in vaginal epithelial cells

**Figure 3:**
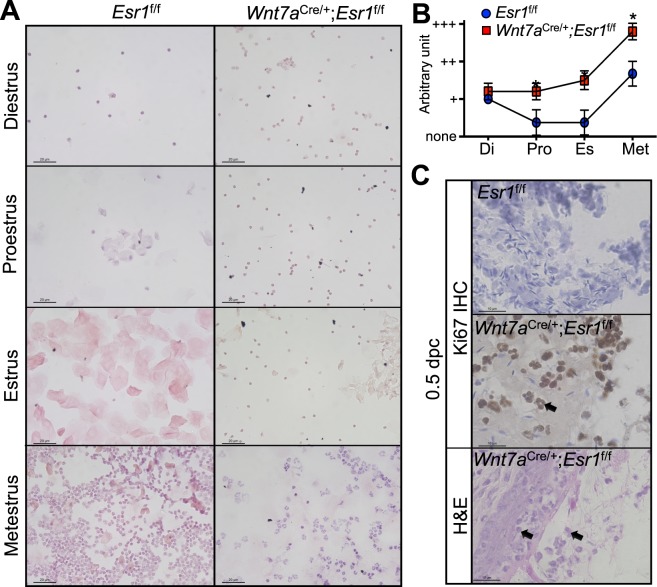
Increased number of leukocytes in the absence of epithelial ESR1. (**A**) H&E staining of vaginal smears from *Esr1*^f/f^ and *Wnt7a*^cre/+^; *Esr1*^f/f^ animals at each stage of estrous cycle. Scale bars = 20 µm. *n* = 3–4 mice/genotype. (**B**) Quantification of leukocyte levels through estrous cycle (arbitrary unit). + = less than 10% of cells within the microscopic field are leukocytes, ++ = 10–60% of cells within the microscopic field are leucocytes, +++ = more than 60% of cells within the microscopic field are leukocytes. Data presented as mean ± SEM. *p < 0.05 compared to *Esr1*^f/f^ at the similar time-point, Mann-Whitney test. *n* = 6 mice/genotype. (**C**) Histological staining of vaginal tissue in *Wnt7a*^cre/+^; *Esr1*^f/f^ compared to *Esr1*^f/f^ animals at 0.5 dpc. Ki67 IHC staining of the vaginal lumen at the top two panels and high magnification of H&E staining of *Wnt7a*^cre/+^; *Esr1*^f/f^ vaginal epithelium at the bottom. Arrows denote multinucleated cells within the semen portion in the vaginal lumen and in the epithelial cell layer. *n* = 4–6 mice/genotype. Scale bar = 10 µm.

Persistent infiltration of leukocytes was one of the most dramatic phenotypes in the *Wnt7a*^cre/+^; *Esr1*^f/f^ vaginal tissue at 0.5 dpc. Therefore, daily vaginal smears were collected to systematically track the estrous cycle of *Esr1*^f/f^ and *Wnt7a*^cre/+^; *Esr1*^f/f^ animals. In *Esr1*^f/f^ animals, leukocyte levels were lowest during proestrus and estrus and highest during metestrus (Fig. 3A,B). However, leukocyte levels were present throughout the entire estrous cycle in *Wnt7a*^cre/+^; *Esr1*^f/f^ animals, resulting in a non-cyclic pattern of leukocyte infiltration (Fig. 3A,B).

**Figure 4:**
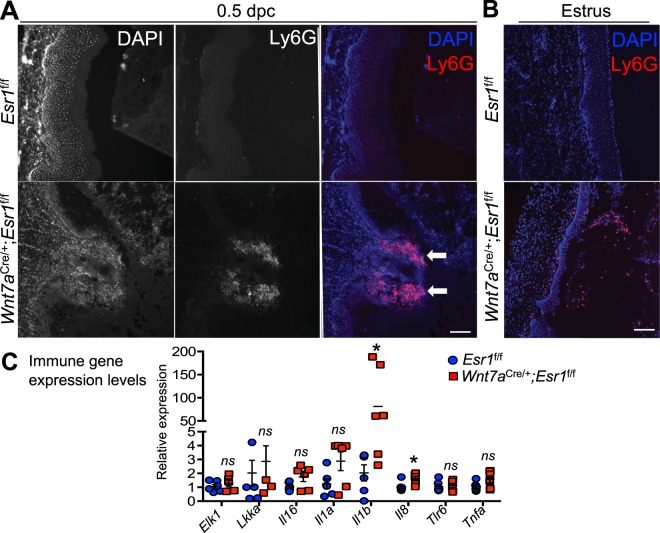
Neutrophil infiltration and upregulation of immune gene expression. DAPI and Ly6G IF staining of *Esr1*^f/f^ and *Wnt7a*^cre/+^; *Esr1*^f/f^ vaginal tissues at (**A**) 0.5 dpc (*n* = 5 mice/genotype) and (**B**) estrus (*n* = 5 mice/genotype). White arrows indicate the protrusion sites in *Wnt7a*^cre/+^; *Esr1*^f/f^ vaginal tissues. (**C**) Immune gene expression levels in *Esr1*^f/f^ and *Wnt7a*^cre/+^; *Esr1*^f/f^ vaginal tissues, measured by RT-qPCR analysis. *n* = 3 mice/genotype. Each data point from all technical replicates are presented. Data presented as mean from each technical replicate ± SEM. *p < 0.05 compared to *Esr1*^f/f^, Mann-Whitney test. *ns* = not statistically significant. Scale bars = 100 µm in (**A** and **B**).

The leukocytes present in the upper vaginal lumen appeared to be multi (or segmented)-nucleated cells, characteristic of neutrophils. In the *Wnt7a*^cre/+^; *Esr1*^f/f^ epithelium, infiltration of the multi-nucleated cells was present within the lumen and in vaginal tissues (Fig. 3C). To validate whether or not the infiltrating cells were neutrophils, Ly6G was used as a neutrophil marker. Ly6G was not detected in the *Esr1*^f/f^ vaginal tissues (Fig. 4A), however, Ly6G-positive cells were detected at lesion sites within the vaginal epithelium of *Wnt7a*^cre/+^; *Esr1*^f/f^ tissues (Fig. 4A). Due to a mating-induced lesion at 0.5 dpc, we evaluated the number of neutrophils present in vaginal tissues at estrus, prior to mating. At estrus, Ly6G-positive cells accounted for 8.0 ± 3.6% of all cells in the epithelial cell layer of *Wnt7a*^cre/+^; *Esr1*^f/f^ tissue compared to the total absence of Ly6G-positive cells in *Esr1*^f/f^ vaginal tissues (Fig. 4B).

To validate whether the lack of a cornified layer alters the expression of immune genes in the vaginal epithelium of *Wnt7a*^cre/+^; *Esr1*^f/f^ animals, we quantified the expression of immune-related genes that were previously reported^9^. Among all tested genes, mRNA levels of *Il1b* and *Il8* were significantly elevated (Fig. 4C, *p* = 0.0152 for both genes), while other immune genes remained unchanged in *Wnt7a*^cre/+^; *Esr1*^f/f^ compared to *Esr1*^f/f^ vaginal tissues.

**Figure 5:**
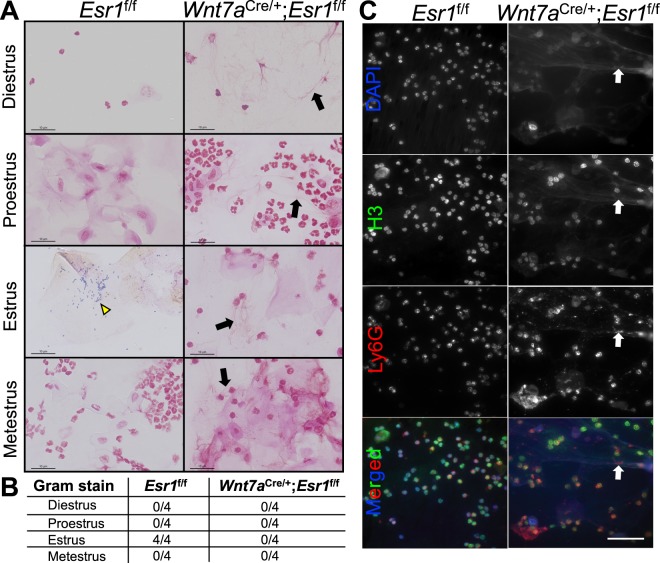
A loss of gram-positive bacteria and increased extruding neutrophil nuclei in vaginal smears from female mice lacking ESR1 in the vaginal epithelial cells. (**A**) Gram-positive staining of vaginal smears over the course of estrous cycle from *Esr1*^f/f^ and *Wnt7a*^cre/+^; *Esr1*^f/f^ animals. Yellow arrowhead indicates gram-positive bacterial colonies. Black arrows indicate extrusion of nuclei in *Wnt7a*^cre/+^; *Esr1*^f/f^ smears. Scale bars = 10 µm. *n* = 4 mice/genotype. (**B**) Number of mice with gram-positive bacterial colonies in their vaginal smear. (**C**) DAPI, H3, and Ly6G staining of vaginal smears from metestrus stage of *Esr1*^f/f^ and *Wnt7a*^cre/+^; *Esr1*^f/f^ animals. White arrows denote neutrophil nuclei with Ly6G-positive staining extruding into the distance. Scale bars = 50 µm. *n* = 5 mice/genotype.

To test whether there is a correlation between the bacterial populations present in the *Wnt7a*^cre/+^; *Esr1*^f/f^ vagina and the increasing number of leukocytes, gram staining was performed on vaginal smears. Colonies of gram-positive bacteria were observed at estrus in all *Esr1*^f/f^ smear samples (Fig. 5A, yellow arrowhead). At metestrus, smears from *Esr1*^f/f^ tissue showed neutrophil infiltration, but gram-positive bacterial colonies were not detected. In smears from diestrus and proestrus stages, we observed scarce or no bacterial colonies. In the *Wnt7a*^cre/+^; *Esr1*^f/f^ animals, gram-positive bacterial colonies were not observed at any stage in any of the animals (Fig. 5A). Bacterial colonies were present only in the smears from *Esr1*^f/f^ at estrus stage, but not *Wnt7a*^cre/+^; *Esr1*^f/f^ females (Fig. 5B). Leukocyte clusters from the vaginal smear of *Wnt7a*^cre/+^; *Esr1*^f/f^ females were too dense to be viewed; therefore, we did not include these areas when screening for gram-positive bacterial colonies (Supplementary Fig. S3). Due to the fiber-like protrusion of the neutrophil nuclei in *Wnt7a*^cre/+^; *Esr1*^f/f^ smears, we investigated whether this protrusion was a result of neutrophil extracellular traps (NETs)^23^ that eliminate surrounding bacteria. Histone 3 (H3) is a critical mediator involved in the formation of NETs^23^, so an H3 antibody was used to determine the presence of NETs in vaginal smear samples. The protrusion pattern from the nuclei region of DAPI and H3 was detected in the *Wnt7a*^cre/+^; *Esr1*^f/f^, but not in *Esr1*^f/f^ smears (Fig. 5C, white arrows). Cells with a protrusion pattern in *Wnt7a*^cre/+^; *Esr1*^f/f^ smear samples also co-localized with Ly6G expression.

### A loss of epithelial ESR1 in the vagina changes enzymatic activities in the cervical-vaginal fluid

**Figure 6:**
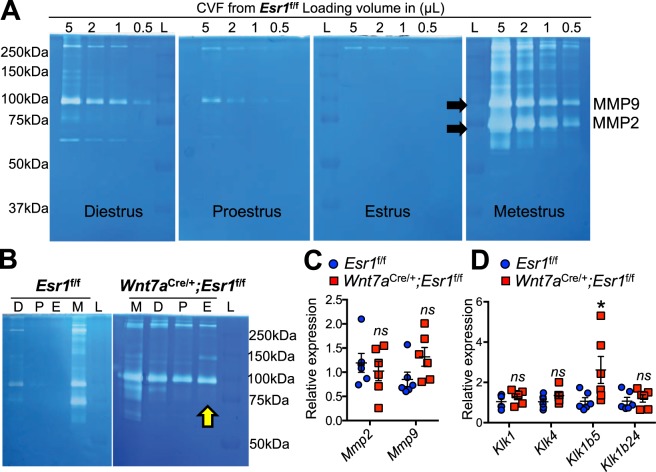
The absence of epithelial ESR1 results in a constant MMP2/9 activity in the cervical-vaginal fluid (CVF). (**A**) Gelatin zymography of CVF from *Esr1*^f/f^ females collected at different stages of the estrous cycle. CVF from *Esr1*^f/f^ females were loaded at different volumes (μL) in the gels. Two major bands (black arrows) are predicted sizes of MMP2 and MMP9, L = protein ladder. *n* = 6 mice/genotype. (**B**) Zymography of CVF from *Esr1*^f/f^ and *Wnt7a*^cre/+^; *Esr1*^f/f^ animals collected at different stages of the estrous cycle. Yellow arrow denotes at the date animal was plugged. D = Diestrus, P = Proestrus, E = Estrus; M = Metestrus. *n* = 6 mice/genotype. (**C**) RT-qPCR of *Mmp2* and *Mmp9* expression levels in *Esr1*^f/f^ and *Wnt7a*^cre/+^; *Esr1*^f/f^ vaginal tissues at estrus *n* = 3 mice/genotype. (**D**) RT-qPCR of vaginal *Klk1*, *Klk4*, *Klk1b5*, and *Klk1b24* expression levels in *Esr1*^f/f^ and *Wnt7a*^cre/+^; *Esr1*^f/f^ vaginal tissues at estrus. *n* = 3 mice/genotype. Data presented as mean from all technical replicate ± SEM. *p < 0.05 compared to *Esr1*^f/f^, *ns* = not statistically significant, Mann-Whitney test. (**A** and **B**) were cropped from four full zymography images presented in Supplementary Fig. S4.

Matrix metalloproteinase 9 (MMP9) is produced by neutrophils that undergo NET transformation in order to induce tissue remodeling^24,25^. To test whether MMPs are produced in *Wnt7a*^cre/+^; *Esr1*^f/f^ cervical-vaginal fluid (CVF), enzymatic activity of CVF was determined using a gelatin zymography assay. In *Esr1*^f/f^ CVF, digestion of the gelatin gel was observed mainly at metestrus (Fig. 6A), corresponding to the leukocyte infiltration. The major bands detected were at 72 and 92 kDa, which are consistent with the size of MMP2 and MMP9, respectively (Fig. 6A). Enzymatic activity of the CVF from *Esr1*^f/f^ females was detected at lower levels during diestrus and proestrus in comparison to metestrus, and not at a detectable level at estrus (Fig. 6A). Persistent MMP digestive activity was evident in *Wnt7a*^cre/+^; *Esr1*^f/f^ CVF when compared to the CVF from *Esr1*^f/f^ controls (Fig. 6B). To assess the relative levels of *Mmp2* and *Mmp9*, RT-qPCR was performed using whole vaginal tissues collected at estrus. We found that *Mmp2* and *Mmp9* mRNA levels were expressed at comparable levels in *Wnt7a*^cre/+^; *Esr1*^f/f^ and *Esr1*^f/f^ tissues (Fig. 6C). In addition to MMPs, other proteases could also induce changes in tissue integrity. In the vagina, we identified four highly expressed *Klk* family members in *Esr1*^f/f^ tissues. Only *Klk1b5* was significantly increased in *Wnt7a*^cre/+^; *Esr1*^f/f^ compared to *Esr1*^f/f^ tissues (Fig. 6D, *p* = 0.0411).

## Discussion

**Figure 7:**
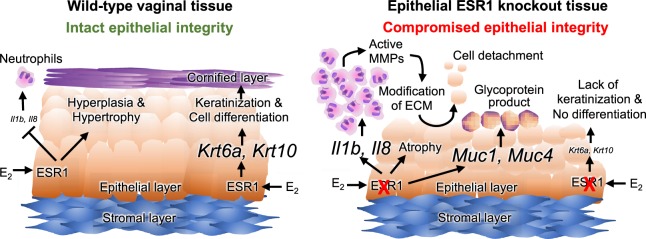
Working model of the function of ESR1 in vaginal epithelial cells. In the presence of epithelial ESR1, E_2_ action through ESR1 suppresses neutrophil infiltration by minimizing the level of cytokine production (*Il1b* and *Il8*) in vaginal tissue. In addition, E_2_ induces epithelial cell hypertrophy and keratin-mediated differentiation via stimulation of *Krt6* and *Krt10* production. A lack of epithelial ESR1 causes excess *Il1b* and *Il8* levels in the vaginal tissue leading to increased recruitment of neutrophils. Elevated neutrophil levels in the vaginal tissues result in excessive MMP activity and subsequent extracellular matrix (ECM) modification. In the absence of ESR1, *Muc1* and *Muc4* levels are increased, causing an overproduction of glycoproteins in the apical membrane. Moreover, the epithelial cells are unable to produce *Krt6a* and *Krt10*, leading to an undifferentiated epithelial layer. These aberrant ECM modifications and undifferentiated epithelial layer leads to a loss of epithelial tissue integrity, and cells are easily detached from the stroma during mating.

Our study revealed new insights regarding the role of epithelial ESR1 in maintaining tissue integrity and immune response in the lower female reproductive tract. We have summarized our findings and proposed a working model in Fig. 7. First, ESR1 in the vaginal epithelium is responsible for E_2_-induced cell hypertrophy during the estrous cycle. Without ESR1, epithelial cells become atrophic, similar to what occurs in postmenopausal women. Second, epithelial ESR1 regulates genes involved in maintaining cellular integrity (keratins) and secretions (mucins). Lack of epithelial ESR1 contributes to a loss of cornification, reduced cellular integrity, and excessive glycoprotein production. Third, epithelial ESR1 plays a vital role in immune suppression. Lacking epithelial ESR1 would result in a failure to suppress vaginal leukocytes, leading to excessive MMP activities. MMPs are proteinases that digest cellular matrix proteins, causing extracellular matrix (ECM) modification and cellular detachment. ECM breakdown may also create a feedback loop to recruit more neutrophils to clear out cellular debris. This proposed working model illustrates that epithelial ESR1 is necessary for homeostasis of the vagina and potentially facilitates the development of treatments for postmenopausal symptoms in women.

Vaginal tissues of *Wnt7a*^cre/+^; *Esr1*^f/f^ animals displayed sites with excessive amounts of immune cells in the different layers of the vaginal tissue and within the lumen after mating. Based on the gross morphology of the cells in the infiltrate of the *Wnt7a*^cre/+^; *Esr1*^f/f^ animals, a majority of the cells appear to have the characteristic of neutrophils (cells with segmented nuclei and cytoplasmic granules) and macrophages (cell with non-segmented nuclei). Ly6G-positive staining confirmed that a majority of the leukocytes were indeed neutrophils. However, further investigation is needed to identify the different immune cell types in the presence or absence of epithelial ESR1 in the vagina. Interestingly, the presence of neutrophils was not reported in the previous *Krt5*^cre/+^; *Esr1*^f/f^ model^4^. At 0.5 dpc, *Wnt7a*^cre/+^; *Esr1*^f/f^ vaginal epithelia showed lesions with neutrophil infiltration. Some lesions were severe, resulting in a complete detachment of the vaginal epithelium. Based on these results, a lack of epithelial ESR1 results in an extremely fragile vaginal epithelium that can be easily damaged by physical stress, such as mating.

Cell atrophy contributes to vaginal dryness and sexual dysfunction in postmenopausal women^21^. Statistical analysis of the epithelial thickness in *Wnt7a*^cre/+^; *Esr1*^f/f^ animals indicates an overall thinning of the epithelium compared to *Esr1*^f/f^ controls. It is established that E_2_ induces cell hypertrophy in the uterus^26^. In this report, a loss of epithelial ESR1 led to a significant reduction in cell size in non-proliferating epithelial cells in *Wnt7a*^cre/+^; *Esr1*^f/f^ at both estrus and 0.5 dpc. This finding suggests that local epithelial ESR1 signaling is necessary to induce vaginal cell hypertrophy.

Cell proliferation is essential for tissue growth. We previously reported that stromal, but not epithelial, ESR1 in the uterus is required for epithelial cell proliferation^22,27^. We speculated that vaginal epithelial cell proliferation is also dependent on stromal ESR1 signals, since the proliferation was indeed independent of epithelial ESR1 expression during estrus. However, an increased proliferation index at 0.5 dpc in *Wnt7a*^cre/+^; *Esr1*^f/f^ vaginal epithelium could be contributed by the Ki67-positive leukocytes in the epithelium. In *Wnt7a*^cre/+^; *Esr1*^f/f^ tissues during estrus, the epithelial cells at the apical side of the lumen were loosely attached to the cells underneath. At 0.5 dpc, epithelial lesions and cellular detachment were evident in *Wnt7a*^cre/+^; *Esr1*^f/f^ tissues. It is highly likely that differentiating cells detached prematurely before they could terminally differentiate into cornified cells, or detached due to physical stress during mating. Based on these observations, a loss of epithelial ESR1 led to a reduced number of non-proliferating cells and an increased proliferating index at 0.5 dpc.

In 1922, Long and Evans described the cyclic pattern of the rat vaginal epithelium^28^. Since then, the process of vaginal keratinization has been studied extensively and determined to be induced by E_2_. Keratin 6, 10, 13, and 16 are involved in the keratinization process^29–33^. In addition to the previous *Krt5*^cre/+^; *Esr1*^f/f^ model, we showed that *Krt*6*a* and *Krt10* were expressed at lower levels in the absence of epithelial ESR1. The expression pattern of keratin is unique to different epithelial cell types as keratins share only 30% sequence homology between different subfamily members^34^. *Krt*6*a* is a type 2 keratin, associated with pachyonychia congenita^35^. *Krt10* is a type 1 keratin and a mutation in *Krt10* is associated with hyperkeratosis^36,37^. Pachyonychia congenita and hyperkeratosis are rare autosomal dominant disorders, and the mutated keratin proteins impair the compaction of the stratum corneum of the skin. Formation of the intermedium filament in the skin requires the dimerization of KRT1 and KRT10^38^. Therefore, reduction in *Krt6a* and *Krt10* expressions may contribute to the absence of the cornified layer and a loss of cellular integrity in the vaginal epithelium.

In contrast to keratinization, vaginal mucification is triggered by changes in circulating levels of P_4_ and E_2_^39,40^. High levels of P_4_ oppose E_2_-induced action resulting in mucification — a production of glycoproteins including mucins. Mucins are the major barrier molecules in the reproductive mucosa. Two members of the mucin family, mucin 1 and 4 are expressed in human vaginal epithelium^41^. Both *Muc1* and *Muc*4 were elevated in the *Wnt7a*^cre/+^; *Esr1*^f/f^ vagina. Moreover, MUC1 protein levels were increased in the upper vaginal tract in the absence of epithelial ESR1. Our findings are consistent with the previous report that suppression of ESR1 activity caused aberrant induction of MUC1 production in the mouse uterus^42^. Thus, we conclude that epithelial ESR1 in the vaginal tissue is required for normal keratinization and mucification processes.

The host defense system of the lower female reproductive tract is composed of epithelial layers, the immune system, vaginal flora, low pH, and mucus in the CVF. The dynamic immune system responds to sex hormones and physical stimuli. In women, elevated E_2_ during the proliferative phase suppresses mucosal immunity, creating a window of vulnerability for infection^6^. In rodents, an increased risk of infection in the lower female reproductive tract is also associated with increased levels of E_2_^43–45^. Our data are consistent with these findings as gram-positive bacterial colonies were present in vaginal smears during the estrus stage of control animals. At estrus, the absence of leukocytes in the vaginal lumen allows the growth of bacteria colonies during this window of vulnerability^6^. In contrast, a loss of epithelial ESR1 caused excessive leukocyte infiltration throughout all stages of the estrous cycle, resulting in a lack of gram-positive bacterial colonies. Thus, it is possible that the ovarian cycle provides a window of recovery in the female reproductive tract, allowing the tissue to grow and normal flora to colonize in an environment with minimal immunosuppression.

In vaginal smears obtained from *Wnt7a*^cre/+^; *Esr1*^f/f^ animals, we observed the presence of viscous material and leukocyte clusters that were similar to vaginal samples obtained from animals with a prolonged diestrus stage, where the mucus entraps neutrophils^10^. Cells within the mucus were distorted or elongated^10^. We identified the majority of the leukocyte population in the CVF from *Wnt7a*^cre/+^; *Esr1*^f/f^ females to be Ly6G-positive neutrophils. It is established that excessive neutrophil activity has a negative impact on tissue health, primarily due to hyperactivity of neutrophil elastase (NE)^46^, a proteinase that degrades epithelial cadherin resulting in a loss of tissue integrity^46^. In the lung, NE reduces respiratory epithelial integrity^46^. In cancer models, neutrophils enhance carcinogenesis and metastatic potential^47,48^. In blood vessels, neutrophil-extravasation increases vascular permeability^49^. In addition, NETs can also cause blood clots in the vessels^50^. However, the mechanism of how excess neutrophils affect vaginal immune system and how E_2_ signaling is involved in this process are unclear. Here, our study showed that *Il1β* and *Il8* were elevated in the absence of epithelial ESR1. This finding is interesting as *Il8* is a proinflammatory cytokine produced by keratinocytes^51^ and is a known neutrophil-activation factor^52,53^. Additionally increased expression of *Il1β* and *Il8* can be triggered by a bacterial infection^54^. The elevated expression of *Il1β* and *Il8* are consistent with their role in neutrophil activation and local inflammatory responses^55,56^.

Activated neutrophils can transform into extracellular traps (NETs) to kill pathogens using dense materials from the cell nucleus^23^. In addition, neutrophils utilize activated MMP9 to induce tissue remodeling^25^. Considering the extensive tissue damage and cell detachment in the *Wnt7a*^cre/+^; *Esr1*^f/f^ vaginal tissue, it is likely that this tissue damage is caused by elevated MMP activity derived from excess neutrophils. Persistent MMP2/9 activity was evident throughout all stages of the estrous cycle in the absence of epithelial ESR1. Immunofluorescent staining showed that nuclei and H3 of the neutrophils were extruding outside of the cell along with strong Ly6G signals. This histological structure of the neutrophils from the CVF of *Wnt7a*^cre/+^; *Esr1*^f/f^ animals matches the description of NETs. These findings suggest that MMP2/9 activity correlates with the presence of neutrophils and is likely modulated by epithelial ESR1 at a post-transcriptional level.

In our previous studies, we identified roles for a different protease family, the kallikreins (KLKs), in the female reproductive tract. KLK expression is tissue-specific. In the oviduct, excessive KLK activity resulted in a lysis of the embryo leading to infertility^1^. In the uterus, a lack of KLK activity caused a semen liquefaction defect^3^. In the vagina, we found that *Klk1b5* was the only KLK member with increased expression in the absence of epithelial ESR1. Therefore, *Klk1b5* remains a unique candidate for our future study as we previously showed its biological function in the upper female reproductive tract.

## Materials and Methods

### Ethics statement

All animal handling protocols and procedures were carried out according to Washington State University (WSU) Animal Care and Use Committee guidelines and were in compliance with WSU-approved animal protocols #4702 and 4735. Studies were performed with mice that were housed in a temperature- and humidity-controlled room with access to water and food *ad libitum*.

### Animals and experimental procedures

Adult female mice (8 to 16 weeks old) with a selective deletion of ESR1 in the epithelial cells of the female reproductive tract (*Wnt7a*^cre/+^; *Esr1*^f/f^) and their control littermates (*Esr1*^f/f^) were used in the experiments. Generation of experimental mice and genotyping of the animals was carried out as previously described^22^. Deletion of epithelial ESR1 in the *Wnt7a*^cre/+^; *Esr1*^f/f^ and *Esr1*^f/f^ experimental animals was confirmed using IHC analysis^3^. *Wnt7a*^cre/+^; *Esr1*^f/f^ and *Esr1*^f/f^ female mice were singly housed and bred overnight with a wild-type (WT) C57BL6/J (The Jackson Laboratory, Bar Harbor, ME) proven breeder male. If a copulatory plug was observed the next morning at 8 a.m., the female was designated as 0.5 days post coitus (0.5 dpc). At the time of tissue collection, animals were euthanized using CO_2_ asphyxiation followed by cervical dislocation.

### Histological procedures

Unless otherwise noted all tissue sections analyzed were obtained from four *Esr1*^f/f^ animals and three *Wnt7a*^cre/+^; *Esr1*^f/f^ animals at estrus stage, and four *Esr1*^f/f^ and six *Wnt7a*^cre/+^; *Esr1*^f/f^ animals at 0.5 dpc. Five-micrometer (µm) paraffin sections were used in this study.

The H&E staining protocol was performed as previously described^3^. In brief, paraffin sections were deparaffinized in two xylene washes (5 mins each, Fisher Chemical), rehydrated in a graded ethanol series (2–3 mins each), stained with hematoxylin (30 secs), rinsed with water (2–3 mins), stained with Eosin (30 secs), dehydrated in a graded ethanol series (2–3 mins each), washes in two xylene washes (5 mins) and mounted in Permount (ThermoFisher Scientific Inc., Carlsbad, CA).

For Masson’s Trichrome staining, paraffin sections were deparaffinized and rehydrated as described above, followed by fixation in Bouin’s solution (75% periodic acid, 10% formaldehyde, 5% acetic acid) at 56 °C for 1 hr to improve the quality of the staining. Sections were then rinsed twice under tap water for 5 mins and stained with Weigert’s iron hematoxylin (0.5% hematoxylin (#0701–50 G, Amresco VWR, Solon, OH) 0.5% hydrochloric acid, 1.2% ferric chloride in 50% ethanol) for 10 mins. Next, sections were stained with Biebrich scarlet-acid Fuchsin (1% Biebrich scarlet, 0.1% acid Fuchsin, and 1% acetic acid) for 10 mins, followed by phosphomolybdic-phosphotungstic acid (2.5% phosphomolybdic acid and 2.5% phosphotungstic acid) for color differentiation. Sections were then washed with distilled water and counterstained with aniline blue (2.5% aniline blue and 2% acetic acid).

For Periodic Acid Schiff staining (PAS), sections were rehydrated as described above and oxidized in 0.5% periodic acid for 5 mins, rinsed, and placed in Schiff’s reagent (Alfa Aesar, Ward Hill, MA) for 15 mins. Sections were then washed in warm tap water for 5 mins and counterstained in Mayer’s hematoxylin for 1 min.

For gram staining, vaginal smears were obtained as previously described^10^. First, smears were air-dried and heat fixed on a gentle flame for 2–3 seconds. Slides were then stained with Gram’s crystal violet (1% crystal violet, 0.5% ammonium oxalate) for 1 min and color-treated with Gram’s Iodine (#470301–188, Ward’s science, Rochester, NY). A 50/50 mixture of 95% ethyl alcohol and acetone was applied as a decolorizer. Slides were then counterstained with 0.5% safranin, washed with tap water, and air dried before mounting with Permount (ThermoFisher Scientific Inc., Carlsbad, CA). Four animals per genotype were used for this experiment.

For IHC staining, the formalin-fixed paraffin-embedded tissues were processed as previously described with minor modifications^3^. Primary antibodies against ESR1 (1:200, #MA5-13191, ThermoFisher Scientific), or Ki67 (1:200, #550609, BD Pharminogen, San Jose, CA), and MUC1 (1:400, #ab15481, abcam, Burlingame, CA) were incubated in 10% Normal Horse Serum (NHS) for 1 hr at room temperature. Mouse IgG was used in place of primary antibodies for a negative control. The secondary antibody (1:1000 biotinylated horse anti-mouse, Vector Laboratories, Burlingame, CA) was applied to the sections for 30 mins. Vectastain RTU Elite and ImmPact kits (Vector Laboratories) were used according to the manufacturer’s directions to detect the positive signals. Tissues were counterstained with hematoxylin, dehydrated, and coverslipped with Permount (ThermoFisher Scientific Inc., Carlsbad, CA).

The immunofluorescence staining protocol was used as previously described with slight modifications^57^. Briefly, cryosections were antigen retrieved with a decloaker (BioCare Medical, Concord, CA), washed in phosphate-buffered saline (PBS) for 10 mins, and blocked with 0.1% triton, and 2% bovine serum albumin (BSA) in PBS for 1 hr at room temperature. Sections were then incubated with histone H3 antibody (1:1000, #AB46765, abcam) at 4 °C overnight. The goat anti-rabbit secondary antibody (1:100, #SA00007-2, ProteinTech, IL, USA) was applied to the sections for 90 mins at room temperature in the dark. After washing, Alexa Fluor 647 anti-mouse Ly6G (1:100, #127609, BioLegend, San Diego, CA) was applied to the sections for 90 mins at room temperature in the dark. Sections were then sealed with ProLong Gold antifade DAPI (#P36935, ThermoFisher Scientific). An Olympus DSU spinning disk confocal microscope was used to capture all fluorescent images. A Leica DMi8 microscope (Leica Microsystems Inc., Buffalo Grove, IL) was used to capture all histological images. Any modifications to images (for example, to increase brightness) were performed across the entire image in accordance with the journal’s standards.

### Mouse estrous cycle stage identification

Examination of vaginal smears stained with H&E were used to determine the estrous cycle stage according to previously described procedures^10^. Vaginal smears were collected at 8:00 am, fixed in methanol (5 mins), and stained with H&E as described above. Vaginal smears with mostly cornified epithelial cells were designated as estrus, the presence of leukocytes and cornified cells were designated as metestrus, smears with very few cells were designated as diestrus, and smears in which the majority of the cells were nucleated were designated as proestrus.

### Reverse Transcriptase-quantitative PCR analysis (RT-qPCR)

The vaginal tract was collected at estrus or at 0.5 dpc for RT-qPCR analysis. Vaginal tissue samples were snap-frozen on dry ice upon removal and stored at −80 °C until use. Tissue samples were homogenized and total RNA was extracted using the RiboZol ME Reagent (Amresco, Solon, OH) according to the manufacturer’s directions. RNA quality and quantity was determined using a NanoDrop 1000 Spectrophotometer (Thermo Scienific). Total RNA (1 μg) was reverse-transcribed using the qScript cDNA SuperMix kit according to the manufacturer’s protocol (Beverly, MA). The cDNA products were diluted 1:5 in nuclease-free H_2_O. Diluted cDNA (1 μL) was used as a template for the RT-qPCR reaction using the PerfeCTa SYBR Green FastMix (QuantaBio) according to the manufacturer’s instructions. PCR reactions were run and raw data was recorded on a 7500 Fast Real-Time PCR System (Applied Biosystems, ThermoFisher Scientific). Expression values in vaginal samples were calculated as fold change and normalized to eukaryotic elongation factor 2 (*Eef2*) expression, relative to the *Esr1*^f/f^. The relative expression of the genes was determined with an *n* = *3* mice/genotype, each of which were measured in triplicate. Primer sequences for the genes analyzed are listed in Supplementary Table S1.

### Cell counting, size measurement, and static analysis

Quantification of Ki67 IHC was determined using with Cell Counter Tool Plugins as previously described^58^. Three images from each tissue section were captured using the Leica Application Suite (Leica Microsystems Inc.). A total of 6 animals per genotype at estrus were used in the analysis with three-to-four consecutive sections cut per animal and used for Ki67 staining. The number of Ki67-positive cells was counted and calculated as the percentage of positive cells in the epithelium in each image as previously described^2^. Cell size was measured using FIJI with Freehands selection and measure tools. A total of 756 cells were counted from four *Esr1*^f/f^ animals at estrus stage, 1039 cells were counted from three *Wnt7a*^cre/+^; *Esr1*^f/f^ animals at estrus stage, 3479 cells were counted from four *Esr1*^f/f^ animals at 0.5 dpc, and 1245 cells were counted from six *Wnt7a*^cre/+^; *Esr1*^f/f^ animals at 0.5 dpc. It is worth noting that the analysis avoided areas of containing lesions due to its abnormal morphology and variability. Only areas where the vaginal epithelium was in contact with the vaginal plug were used in the analysis to ensure consistency in location and cell compositions, across all analyzed animals.

### Cervical-Vaginal Fluid collection and zymography

To collect the CVF, the vaginal canal was flushed with 80 μl of normal saline using P1000 pipet tip. To determine the stage of the estrous cycle, 10 μl of the CVF was aliquoted for H&E staining. The rest of the CVF was stored at −80 °C until use. A total of 6 females/genotype were used in the zymography assay. To remove cell debris, the CVF was centrifuged at 3000 × *g* for 2 mins and the supernatant was used for zymography. Acrylamide gelatin gel (10%) was used for the zymography assay as previously described^59^. In short, CVF samples were incubated with 2X Laemmli sample buffer (#161–0737, Bio-Rad, Hercules, CA) without reducing agents for 10 mins on ice. Then the samples were directly loaded into the gel. After separation of the band at 200 V for 90 mins, the gel was removed from the glass cassette and incubated for 1 hr at room temperature on a shaker with 2.5% Triton X-100 added to the wash buffer (50 mM Tris pH7.4, 5 mM CaCl_2_, 1 μM ZnCl_2_) to remove sodium dodecyl sulfate. The gel was then washed with deionized water and placed in a wash buffer at 37 °C for 20 hrs. Then, the gel was stained with Coomassie stain (2.5% Coomassie G250, 30% Ethanol, 10% acetic acid) for 30 mins. To obtain clear digested bands, the gel was destained for 45 mins with 30% ethanol and 10% acetic acid. To stop the destaining process, the gel was incubated with 2% acetic acid. The visible bands on the gel were captured using a DLSR camera (Canon Rebel T3i) under room lighting, Original images were used in the figures without any editing through image processing software except cropping was performed in ImageJ.

### Statistical analysis

Statistical analysis was performed using GraphPad (Prism, La Jolla, CA) and all data are presented as mean ± standard error of the mean (SEM). The Mann-Whitney post-hoc test was performed on cell and genetic analyses. A two-way ANOVA was performed when data was compared between time points and genotypes unless otherwise indicated.

## Electronic supplementary material

Supplementary Information

## References

[1] Winuthayanon W (2015). Oviductal estrogen receptor alpha signaling prevents protease-mediated embryo death. Elife.

[2] Li S (2017). Estrogen receptor alpha is required for oviductal transport of embryos. FASEB J.

[3] Li S, Garcia M, Gewiss RL, Winuthayanon W (2017). Crucial role of estrogen for the mammalian female in regulating semen coagulation and liquefaction *in vivo*. Plos Genet.

[4] Miyagawa S, Iguchi T (2015). Epithelial estrogen receptor 1 intrinsically mediates squamous differentiation in the mouse vagina. Proc Natl Acad Sci USA.

[5] Wira CR, Grant-Tschudy KS, Crane-Godreau MA (2005). Epithelial cells in the female reproductive tract: a central role as sentinels of immune protection. Am J Reprod Immunol.

[6] Wira CR, Rodriguez-Garcia M, Patel MV (2015). The role of sex hormones in immune protection of the female reproductive tract. Nat Rev Immunol.

[7] Amjadi F, Salehi E, Mehdizadeh M, Aflatoonian R (2014). Role of the innate immunity in female reproductive tract. Adv Biomed Res.

[8] Patel MV, Fahey JV, Rossoll RM, Wira CR (2013). Innate immunity in the vagina (part I): estradiol inhibits HBD2 and elafin secretion by human vaginal epithelial cells. Am J Reprod Immunol.

[9] Wagner RD, Johnson SJ (2012). Probiotic lactobacillus and estrogen effects on vaginal epithelial gene expression responses to Candida albicans. J Biomed Sci.

[10] Cora MC, Kooistra L, Travlos G (2015). Vaginal Cytology of the Laboratory Rat and Mouse: Review and Criteria for the Staging of the Estrous Cycle Using Stained Vaginal Smears. Toxicol Pathol.

[11] Hubscher CH, Brooks DL, Johnson JR (2005). A quantitative method for assessing stages of the rat estrous cycle. Biotech Histochem.

[12] Hickey DK, Fahey JV, Wira CR (2013). Mouse estrous cycle regulation of vaginal versus uterine cytokines, chemokines, alpha-/beta-defensins and TLRs. Innate Immun.

[13] Salamonsen LA, Hannan NJ, Dimitriadis E (2007). Cytokines and chemokines during human embryo implantation: roles in implantation and early placentation. Semin Reprod Med.

[14] Wira CR, Fahey JV, Sentman CL, Pioli PA, Shen L (2005). Innate and adaptive immunity in female genital tract: cellular responses and interactions. Immunol Rev.

[15] Ochiel DO, Fahey JV, Ghosh M, Haddad SN, Wira CR (2008). Innate Immunity in the Female Reproductive Tract: Role of Sex Hormones in Regulating Uterine Epithelial Cell Protection Against Pathogens. Curr Womens Health Rev.

[16] Hickey DK, Patel MV, Fahey JV, Wira CR (2011). Innate and adaptive immunity at mucosal surfaces of the female reproductive tract: stratification and integration of immune protection against the transmission of sexually transmitted infections. J Reprod Immunol.

[17] Carson DD (1998). Mucin expression and function in the female reproductive tract. Hum Reprod Update.

[18] Moncla BJ, Chappell CA, Debo BM, Meyn LA (2016). The Effects of Hormones and Vaginal Microflora on the Glycome of the Female Genital Tract: Cervical-Vaginal Fluid. Plos One.

[19] Muhleisen AL, Herbst-Kralovetz MM (2016). Menopause and the vaginal microbiome. Maturitas.

[20] Hummelen R (2011). Vaginal microbiome and epithelial gene array in post-menopausal women with moderate to severe dryness. Plos One.

[21] Lethaby, A., Ayeleke, R. O. & Roberts, H. Local oestrogen for vaginal atrophy in postmenopausal women. *Cochrane Database Syst Rev*, CD001500 (2016). 10.1002/14651858.CD001500.pub3 PMC7076628 27577677

[22] Winuthayanon W, Hewitt SC, Orvis GD, Behringer RR, Korach KS (2010). Uterine epithelial estrogen receptor alpha is dispensable for proliferation but essential for complete biological and biochemical responses. Proc Natl Acad Sci USA.

[23] Brinkmann V (2004). Neutrophil extracellular traps kill bacteria. Science.

[24] Snelgrove RJ (2010). A critical role for LTA4H in limiting chronic pulmonary neutrophilic inflammation. Science.

[25] Mantovani A, Cassatella MA, Costantini C, Jaillon S (2011). Neutrophils in the activation and regulation of innate and adaptive immunity. Nat Rev Immunol.

[26] Martin L, Finn CA, Trinder G (1973). Hypertrophy and hyperplasia in the mouse uterus after oestrogen treatment: an autoradiographic study. J Endocrinol.

[27] Winuthayanon W (2017). Juxtacrine Activity of Estrogen Receptor alpha in Uterine Stromal Cells is Necessary for Estrogen-Induced Epithelial Cell Proliferation. Sci Rep.

[28] Long, J. A. & Evans, H. M. *The Oestrous Cycle in the Rat and Its Associated Phenomena*, (University of California Press, 1992).

[29] Gimenez-Conti IB (1994). Expression of keratins in mouse vaginal epithelium. Differentiation.

[30] Rotty JD, Coulombe PA (2012). A wound-induced keratin inhibits Src activity during keratinocyte migration and tissue repair. J Cell Biol.

[31] Chateau D, Boehm N (1996). Regulation of differentiation and keratin 10 expression by all-trans retinoic acid during the estrous cycle in the rat vaginal epithelium. Cell Tissue Res.

[32] Schaller G, Lengyel E, Pantel K, Hardt W, Mischke D (1993). Keratin expression reveals mosaic differentiation in vaginal epithelium. Am J Obstet Gynecol.

[33] Chen B (2006). Microarray analysis of differentially expressed genes in vaginal tissues from women with stress urinary incontinence compared with asymptomatic women. Hum Reprod.

[34] Loschke F, Seltmann K, Bouameur JE, Magin TM (2015). Regulation of keratin network organization. Curr Opin Cell Biol.

[35] Bowden PE (1995). Mutation of a type II keratin gene (K6a) in pachyonychia congenita. Nat Genet.

[36] Chen PJ (2016). S159P mutation of keratin 10 gene causes severe form of epidermolytic hyperkeratosis. J Eur Acad Dermatol Venereol.

[37] Mirza H (2015). Mutations Affecting Keratin 10 Surface-Exposed Residues Highlight the Structural Basis of Phenotypic Variation in Epidermolytic Ichthyosis. J Invest Dermatol.

[38] Wallace L, Roberts-Thompson L, Reichelt J (2012). Deletion of K1/K10 does not impair epidermal stratification but affects desmosomal structure and nuclear integrity. J Cell Sci.

[39] Klein M (1937). The Mucification of the Vaginal Epithelium in Rodents. Proceedings of the Royal Society of London. Series B, Biological Sciences.

[40] Meyer RK, Allen WM (1932). The Production of Mucification of the Vaginal Epithelium of Rodents by the Oestrous Hormone. Science.

[41] Gipson IK (1997). Mucin genes expressed by human female reproductive tract epithelia. Biol Reprod.

[42] Lee DK (2010). Suppression of ERalpha activity by COUP-TFII is essential for successful implantation and decidualization. Mol Endocrinol.

[43] Fidel PL, Cutright J, Steele C (2000). Effects of reproductive hormones on experimental vaginal candidiasis. Infect Immun.

[44] Taylor-Robinson D, Furr PM, Hetherington CM (1990). Neisseria gonorrhoeae colonises the genital tract of oestradiol-treated germ-free female mice. Microb Pathog.

[45] Kita E, Takahashi S, Yasui K, Kashiba S (1985). Effect of estrogen (17 beta-estradiol) on the susceptibility of mice to disseminated gonococcal infection. Infect Immun.

[46] Boxio R (2016). Neutrophil elastase cleaves epithelial cadherin in acutely injured lung epithelium. Respir Res.

[47] Gaida MM (2012). Polymorphonuclear neutrophils promote dyshesion of tumor cells and elastase-mediated degradation of E-cadherin in pancreatic tumors. Eur J Immunol.

[48] Fu H (2016). Persisting and Increasing Neutrophil Infiltration Associates with Gastric Carcinogenesis and E-cadherin Downregulation. Sci Rep.

[49] Wessel F (2014). Leukocyte extravasation and vascular permeability are each controlled *in vivo* by different tyrosine residues of VE-cadherin. Nat Immunol.

[50] Jimenez-Alcazar M (2017). Host DNases prevent vascular occlusion by neutrophil extracellular traps. Science.

[51] Feldmeyer L (2007). The inflammasome mediates UVB-induced activation and secretion of interleukin-1beta by keratinocytes. Curr Biol.

[52] Hoffmann E, Dittrich-Breiholz O, Holtmann H, Kracht M (2002). Multiple control of interleukin-8 gene expression. J Leukoc Biol.

[53] Lindley I (1988). Synthesis and expression in Escherichia coli of the gene encoding monocyte-derived neutrophil-activating factor: biological equivalence between natural and recombinant neutrophil-activating factor. Proc Natl Acad Sci USA.

[54] Kistowska M (2014). IL-1beta drives inflammatory responses to propionibacterium acnes *in vitro* and *in vivo*. J Invest Dermatol.

[55] Warnatsch A, Ioannou M, Wang Q, Papayannopoulos V (2015). Inflammation. Neutrophil extracellular traps license macrophages for cytokine production in atherosclerosis. Science.

[56] Cho JS (2012). Neutrophil-derived IL-1beta is sufficient for abscess formation in immunity against Staphylococcus aureus in mice. Plos Pathog.

[57] Li S (2015). DSCAM promotes refinement in the mouse retina through cell death and restriction of exploring dendrites. J Neurosci.

[58] Schindelin J (2012). Fiji: an open-source platform for biological-image analysis. Nat Methods.

[59] Toth M, Sohail A, Fridman R (2012). Assessment of gelatinases (MMP-2 and MMP-9) by gelatin zymography. Methods Mol Biol.

