# Supplementary material for: Estrogen Action in the Epithelial Cells of the Mouse Vagina Regulates Neutrophil Infiltration and Vaginal Tissue Integrity
Source: Sci Rep. 2018 Jul 26;8:11247. doi: 10.1038/s41598-018-29423-5 (PMC6062573; doi:10.1038/s41598-018-29423-5)
Supplement: Supplementary file 1 — Supplementary Information [file 41598_2018_29423_MOESM1_ESM.docx]

**Supplementary Materials**

**Title:** Estrogen Action in the Epithelial Cells of the Mouse Vagina Regulates Neutrophil Infiltration and Vaginal Tissue Integrity

**Authors:** Shuai Li, Gerardo G. Herrera, Keila K. Tam, Jacob S. Lizarraga, My-Thanh Beedle, Wipawee Winuthayanon

**Supplemental Figures**

**
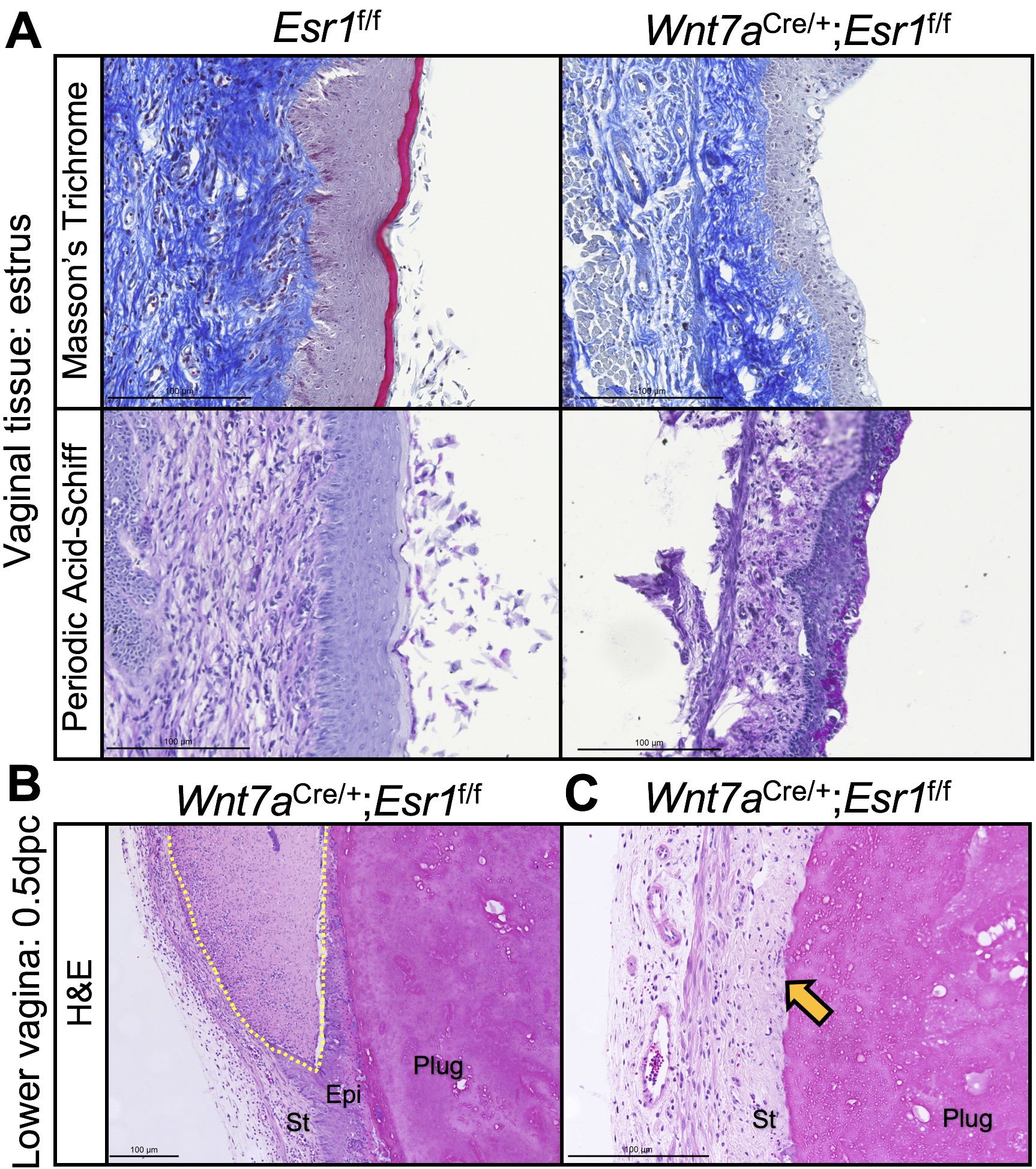
**

**Figure S1**. Representative images of H&E staining shows severe lesions of *Wnt7a*^cre/+^;*Esr1*^f/f^ animals. A) Masson’s Trichrome staining and PAS staining of the vaginal tract at estrus in *Esr1*^f/f^ and *Wnt7a*^cre/+^;*Esr1*^f/f^ animals. B) Mating induced lesion created a cavity allowing semen to fill between the upper and the lower layers of *Wnt7a*^cre/+^;*Esr1*^f/f^ epithelium (yellow dotted lines outline the cavity). C) An area with complete removal of the epithelium (arrow denotes where the vaginal plug adheres to the stromal cells). Scale bars are 100µm.


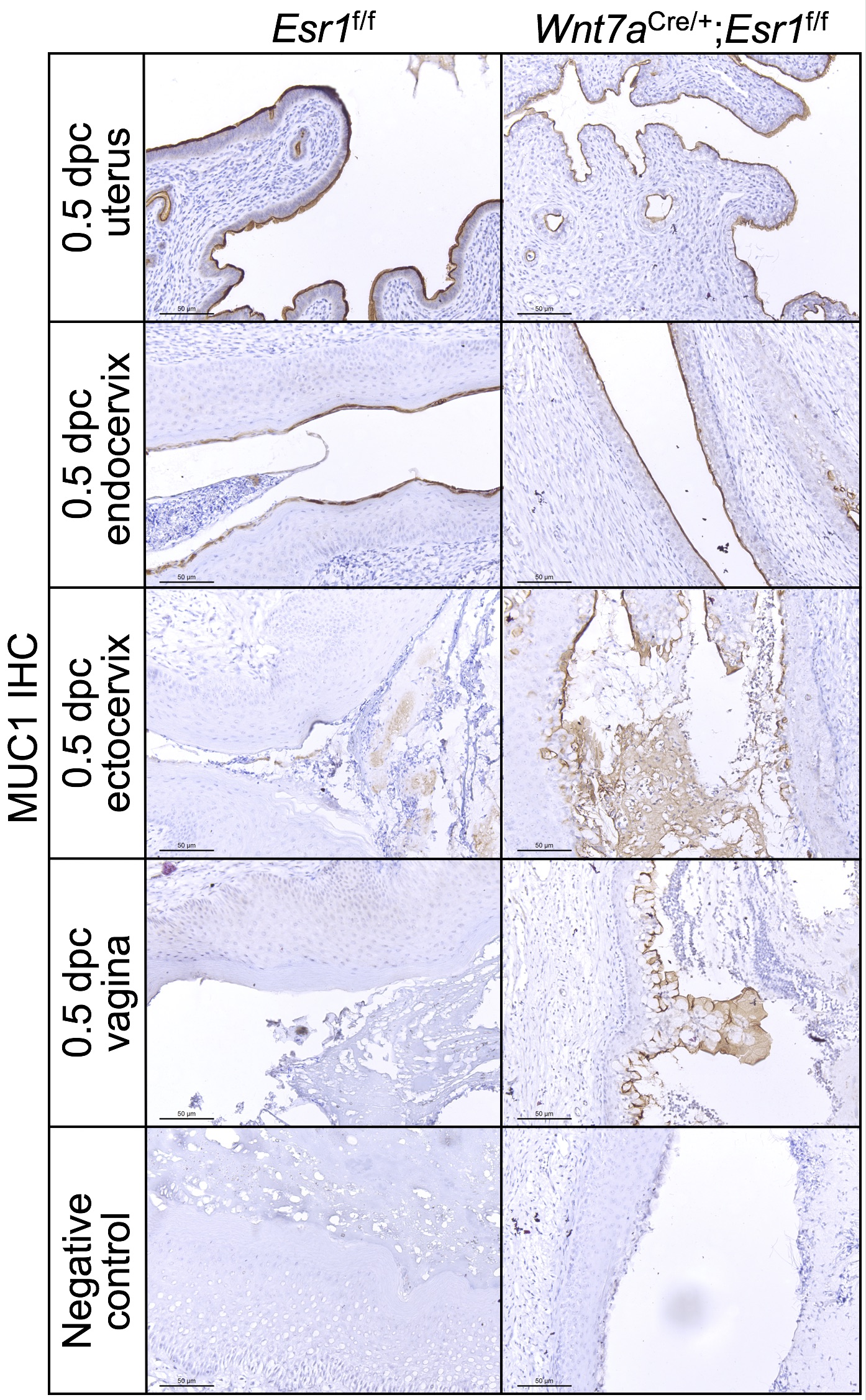


**Figure S2**. Representative images of MUC1 IHC staining of the female reproductive tract. The top panel: MUC1 IHC demonstrates a strong positive staining at the apical layer of the uterine epithelial cells in both *Esr1*^f/f^ and *Wnt7a*^Cre/+^;*Esr1*^f/f^ animals. The second panel: MUC1 was present at the apical surface of endocervix region in both *Esr1*^f/f^ and *Wnt7a*^Cre/+^;*Esr1*^f/f^ animals. The third and fourth panels: MUC1 IHC staining was not detected at the ectocervix or the vagina in *Esr1*^f/f^ animals, but the intense signal was observed at the ectocervix and the upper to middle vaginal tract of *Wnt7a*^Cre/+^;*Esr1*^f/f^ animals. Bottom panel: negative controls omitting primary antibody. Scale bars are 50µm.

**
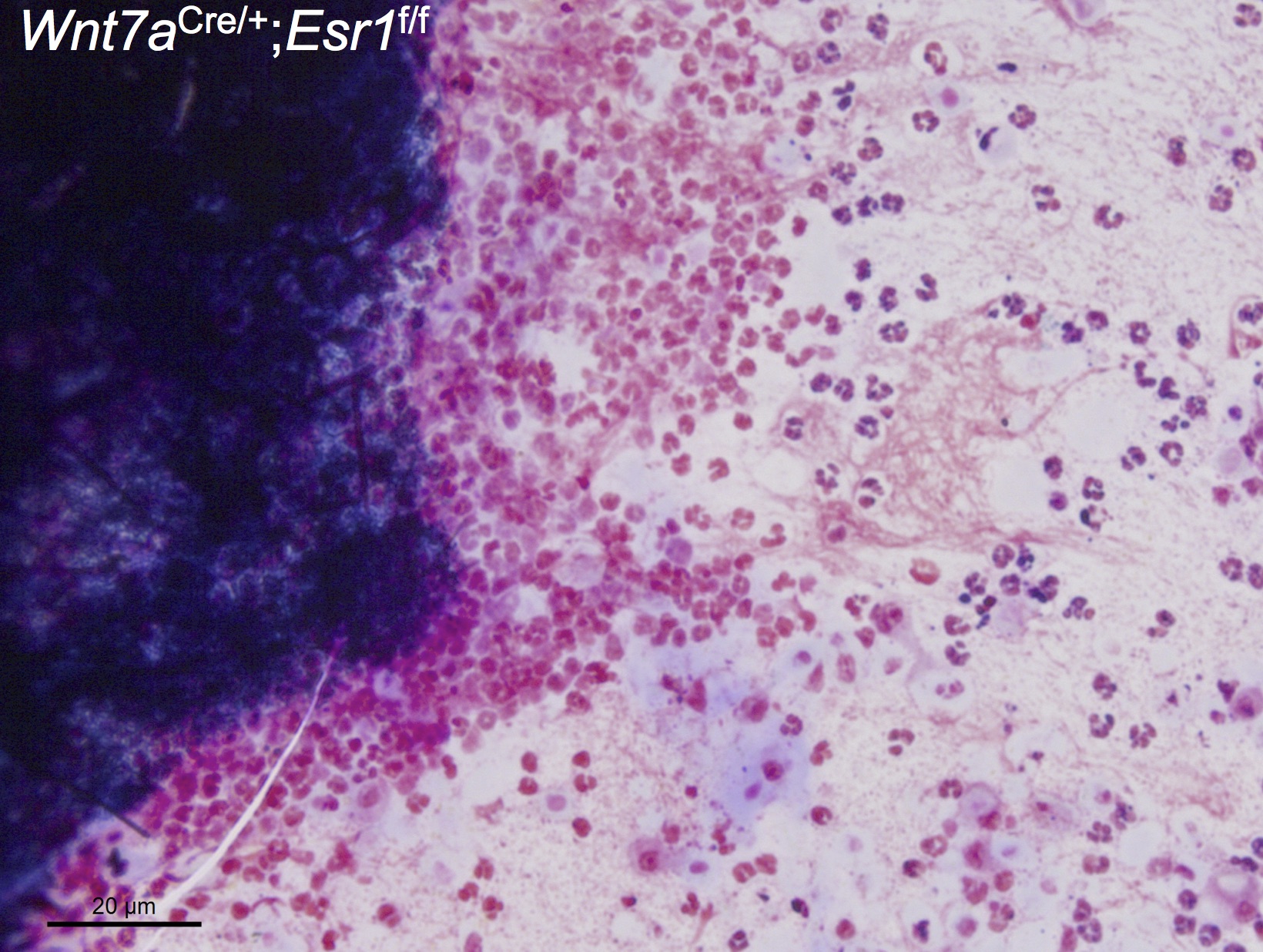
**

**Figure S3**. An example of a leukocyte cluster that was commonly found in the vaginal smears of *Wnt7a*^cre/+^;*Esr1*^f/f^ animals Scale bar is 20µm.

**
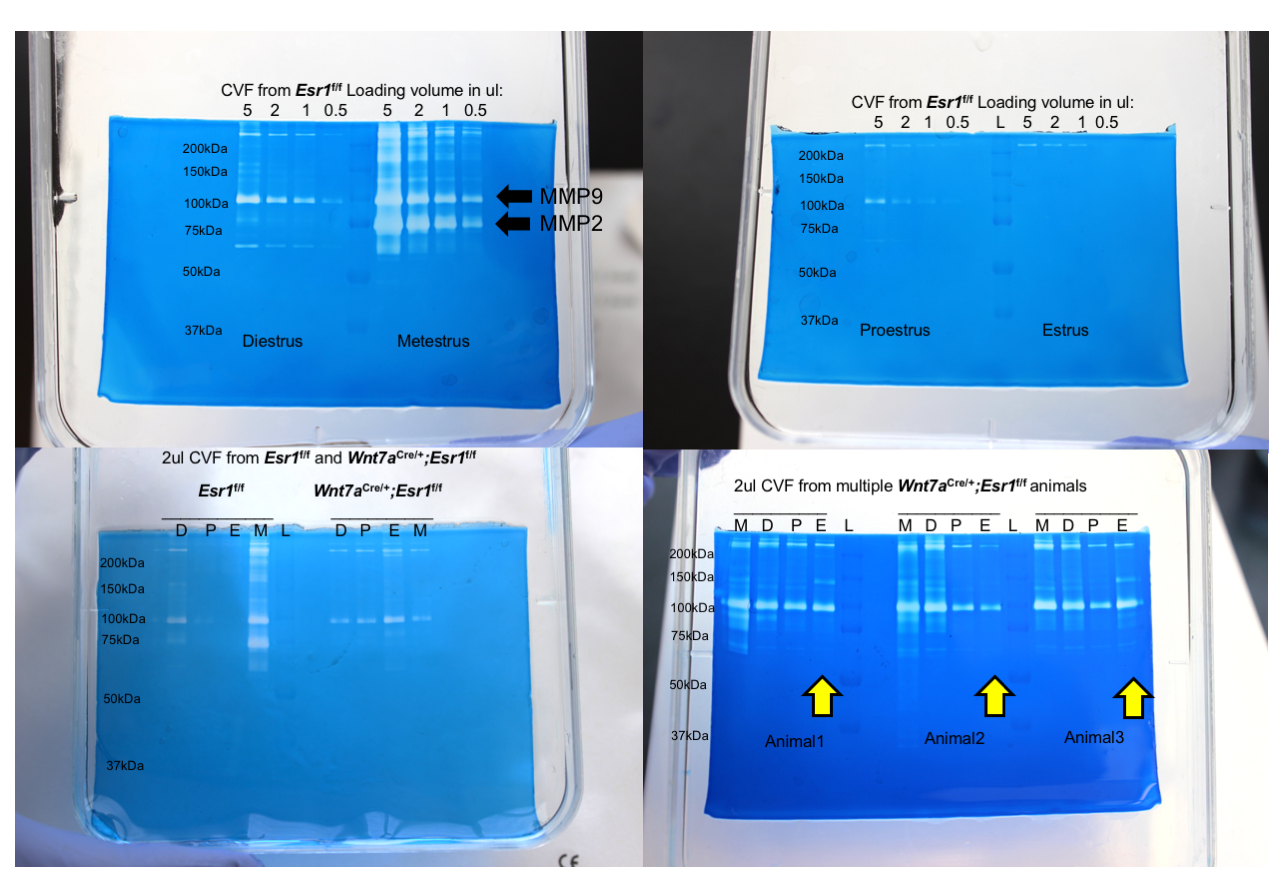
**

**Figure S4**. Original zymography images. Images were not altered by any image processing software. Fig. 6**A** and 6**B** were cropped from the representative areas of above images and were rearranged to improve the clarity of the paper.

**Supplementary Table**

**Table S1**. Primers used for qRT-PCR.

| **Gene names** | **Primer sequences** |
| --- | --- |
| *mMuc1* | F: CCCCTATGAGGAGGTTTCGG  R: CAGATCAGAGTGCAGGGGTC |
| *mMuc4* | F: GACCAGATGGCTCTGAACCTA  R: TGCATTGGCCTCCATTGTGA |
| *mKrt6a* | F: TTCGTGACCCTGAAGAAGGAT  R:TTGCATCTGAGACAGTTCTGCTT |
| *mKrt6b* | F: GAAAAAGGATGTAGATGCTGCC  R: AGGAAGTTGATCTCGTCTGCAA |
| *mKrt10* | F: CAGCTGGCCCTGAAACAATC  R: AGTTGTTGGTACTCGGCGTT |
| *mKrt13* | F: AGAGCGGGACTACAGTGCTT  R:AGCTCATTTTCGTACTTGAGCCTG |
| *mKrt16* | F: ATGGCGAGAATATCCACAGCTC  R: GCTGGTTGAACCTTGCTCCT |
| *mMmp9* | F: GCCGACTTTTGTGGTCTTCC  R: GGTACAAGTATGCCTCTGCCA |
| *mMmp2* | F: AACGGTCGGGAATACAGGAG  R: GGTAAACAAGGCTTCATGGGG |
| *mEef2* | F: CGCTACCTGGCCGAAAAGTA  R: GTACTGCACACCCTTGGTGA |
| *mElk1* | F: CCTGCAGGTTATCCTAACCCC  R: GCCCTTGCAGCTTCCAATTC |
| *mlkka* | F: AACATCCTCTGACATGTGTGGT  R:CCAACTCCAATCAAGACTCATCA |
| *mIl1a* | F: CGTGTTGCTGAAGGAGTTGC  R: GGTGCACCCGACTTTGTTCT |
| *mIl1b* | F: AGCTTCCTTGTGCAAGTGTC  R: TGGGGTCCGTCAACTTCAAA |
| *mIl6* | F: CCTCTCTGCAAGAGACTTCCAT  R: ACAGGTCTGTTGGGAGTGGT |
| *mIl8* | F: GGAAGTGATAGCAGTCCCAAA  R: AATTGGGCCAACAGTAGCCT |
| *mTlr2* | F: AACCTCAGACAAAGCGTCAA  R: TCCTGAGCAGAACAGCGTTT |
| *mTlr6* | F: GCTGTTTTGGGCCAACCTTAG  R: ATGAGAGCCCAGGTTGACAGT |
| *mTnfa* | F: ACTGAACTTCGGGGTGATCG  R: GGTGGTTTGTGAGTGTGAGG |
| *mKlk1* | F: GCCCACTGATCTGTGATGGT  R: TCTGGTGTAGATACCCGGCA |
| *mKlk4* | F: GCTGCTGTATGACCCTGTGT  R: CAGAGTCCCCATTGCAGGAG |
| *mKlk1b3* | F: TCCCTAGGAGGGATTGATGCT  R: CACATGCCAGGGTTGGGAAT |
| *mKlk1b5* | F: ACCCGTCATATACGAACCCG  R: GGGCTTTGACACAGTCCTCA |
| *mKlk1b24* | F: CAACGTGAACCCTCTGCTCA  R: CCTTAGGTTGCGGGATGTCA |
